# Vascular Complications following Vacuum-Assisted Breast Biopsy (VABB): A Case Report and Review of the Literature

**DOI:** 10.3390/tomography9040099

**Published:** 2023-06-24

**Authors:** Ernesto Pansa, Giuseppe Guzzardi, Silvia Santocono, Alessandro Carriero

**Affiliations:** 1 Specializzando in Radiodiagnostica UPO, 28100 Novara, Italy; 2 Dipartimento di Medicina traslazionale UPO, 28100 Novara, Italy; 3 Radiologia AOU Maggiore della Carità, 28100 Novara, Italy

**Keywords:** vacuum-assisted breast biopsy, vabb, hematoma, embolization, case report, protocol, emergency

## Abstract

Introduction: Vacuum-assisted breast biopsy (VABB) has been evaluated as a minimally invasive, safe, and accurate procedure with low complication risks; the most frequent one is the mild/moderate hematoma, which occurs with a low-frequency rate, and the majority of patients who experienced it can be treated successfully with only manual compression and dressing. Although cases of uncontrollable catastrophic bleeding are exceedingly rare, local breast vessel involvement is a concrete risk, even in patients with no bleeding propensity. Case Presentation: In this article, we aimed to describe a 60 years-old woman who, following VABB, experienced a massive hematoma without external bleeding and was successfully treated with embolization. The woman was called back for a cluster of suspicious microcalcifications identified in the left breast’s upper-outer quadrant; however, following histopathological analysis, the few samples collected were negative. She had a silent past medical history, 100% performance status, and no active pharmacotherapy. Approximately 15–30 min after VABB, the patient complained of weakness, pain, and lipothymia. A physical examination revealed a massive hematoma without external bleeding. Clinical data reported PaO 65/40 mmHg and blood chemistry Hb < 10 g/dL. The emergency team was alerted to stabilize the patient, and after that, the breast hemorrhage was controlled by endovascular embolization. Despite this being a rare occurrence, it is important to draw up and follow an appropriate protocol to ensure proper patient management and early treatment. Discussion: This case illustrates the prompt and accurate management of a rare complication following VABB. Due to the very high number of patients undergoing this particular procedure, we aim to point out the concrete risk of vascular injury; other similar cases are described to support our thesis and provide different clinical manifestations of this rare occurrence.

## 1. Introduction

Breast cancer is the most common malignancy disease worldwide. According to a 2019 systematic review and meta-analysis by the Medical School of Zhejiang University, a total of about 1.7 million people were diagnosed with this disease in 2012 worldwide, and 521,900 patients died from it [1,2,3,4].

Breast cancer incidences were high in Western (0.960%) and Northern European countries (0.916%), North America (0.894%), and Australia/New Zealand (0.858%); mortality rates were high in West (0.201%) and Northern (0.174%) and Central and Eastern Europe (0.165%).

A more recent statistical analysis has confirmed breast cancer to be the one characterized by the highest incidence [5].

Therefore, prevention programs have been developed in order to reduce the mortality of this pathology. However, the recommendations about screening age, methods, and intervals have varied with different guidelines due to different institution-based evidence and development processes [6]. In general, most guidelines have recommended annual or biennial mammographic screening for average-risk populations aged between 40 and 74 years and early annual mammography or annual magnetic resonance imaging for high-risk populations.

Early diagnosis is important to decrease the number of deaths and improve prognosis. Broeders M et al. evaluated that a reduction in breast cancer mortality associated with mammographic population-based service screening programs is in the range of 25–31% for women invited for screening and 38–48% for women actually screened with a sufficient follow-up time [7]. More recent data support the positive effects of breast cancer screening [8,9].

Mammographic screening also increased the number of patients who underwent a biopsy.

The potential concerns for mammographic screening include a very small risk of inducing breast cancer from radiation exposure and the risks of overdiagnosis, including anxiety from false positives and unnecessary biopsies.

Soren Cr et al. [10] pointed out how the benefit-to-harm balance of screening mammography could be expected to increasingly tilt towards the harms, and they expected this deterioration in the benefit-to-harm ratio to continue due to an ongoing improvement in therapies.

The continuous development of increasingly safer techniques has made it possible to minimize the risks associated with further investigations, but also a re-evaluation of national screening programs in high-income countries may be needed.

Among recently developed minimally invasive techniques, VABB, which appeared in 1995, can be used as a pre-operative diagnostic procedure and for the treatment of benign and early-stage breast lesions < 2 cm [11,12]; however, the most recent data do not support its efficacy as an alternative treatment compared to standard surgery, even for lesions < 2 cm [13,14,15].

VABB is usually performed under ultrasonography or mammography guidance with a variable duration of between 15–60 min. Most adverse events are, in order of frequency, hematoma, bleeding, local pain, vasovagal reflex, and infection [4]; in this report, we describe a case of vascular complication that was successfully treated with emergency embolization.

The literature underlines the safety of VABB, signaling that the most serious complications are very rare; however, it is important to highlight the worst-case scenario as reported by us and by other case reports.

## 2. Case Report

A 60-year-old woman was called back for additional imaging by the screening program due to suspicious microcalcifications identified in the left breast’s upper-outer quadrant; tomosynthesis and an ultrasound examination were performed and confirmed the microcalcifications detected in the standard projections and initially categorized BI-RADS 3 [16]. After 6 months (short interval follow-up), the cluster increased, and the patient was called back for further evaluation.

VABB under mammography guidance was scheduled, and routine blood chemistry was requested to quantify the risk of bleeding.

Clinical data:

○ Silent past medical history.
○ Normal blood chemistry (in particular, hemoglobin, fibrinogen, platelets, prothrombin time, partial thromboplastin time)
○ No active pharmacotherapy.
○ No allergies were reported.

The patient (60 years old) had a very low risk of bleeding and a Karnofsky [17] performance score of 100%. First of all, the patient’s clinical-anamnestic data, blood chemistry, and informed consent were verified, and subsequently, a peripheral venous catheter on the basilic vein of the right upper limb was positioned. Stereotactic biopsy tables can be prone or seated upright. We performed VABB in a prone position. The advantage of a prone table is the elimination of risk from vasovagal syncope. The seated stereotactic apparatus is less expensive and may offer greater patient comfort. The breast is immobilized in compression during the entire procedure.

**Figure 1:**
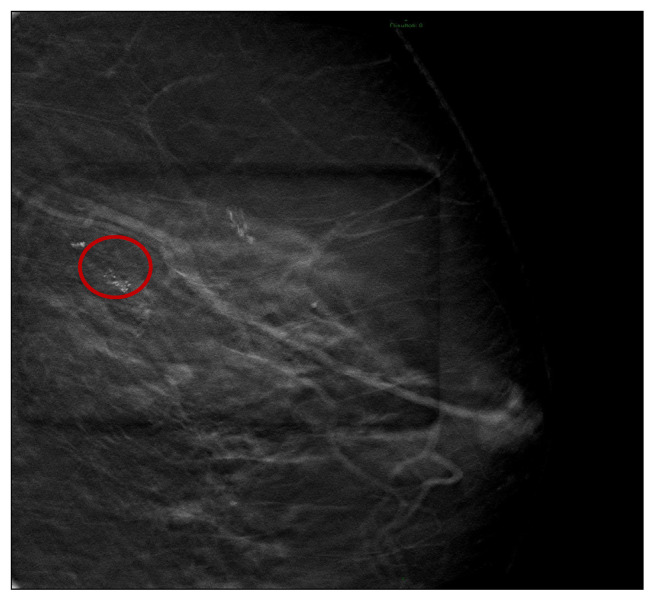
Cluster of microcalcifications detected. (Red circle).

**Figure 2:**
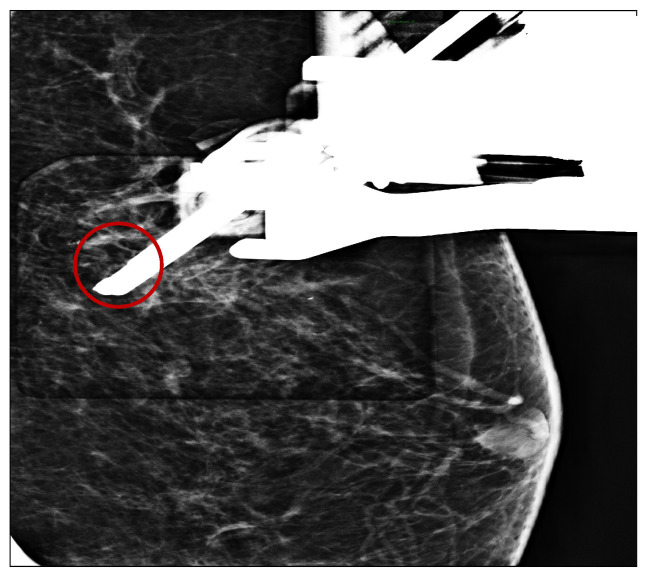
VABB positioned in the microcalcifications spot. (Red circle).

After the administration of a local anesthetic (5 cc of lidocaine + adrenaline 20 mg/mL + 5 mcg; adrenaline induces local vasoconstriction by decreasing bleeding), four tissue samples were taken under mammography guidance VABB with an 11 gauge needle in the microcalcification’s area located in the upper-outer quadrant (Figure 1 and Figure 2).

Thereafter, the procedure was suspended due to bleeding, which was resolved spontaneously with compression and dressing.

The samples collected were sent to the Pathological Anatomy Unit for relative analysis.

The patient was put under observation for 60 min according to our protocol.

Approximately 15–30 min after, the patient reported weakness, pain, and lipothymia.

A physical examination and inspection of the dressing revealed extensive swelling of the left *corpus mammae* and voluminous asymmetry of the breasts without external bleeding.

Clinical data: PaO 65/40 mmHg and BC Hb < 10 g/dL.

The emergency team was alerted, and endovascular embolization was performed after the patient’s stabilization. The procedure was performed in an emergency, under local anesthesia and anesthetist assistance, with right common transfemoral access (5F) and the selective catheterization of the left subclavian artery.

**Figure 3:**
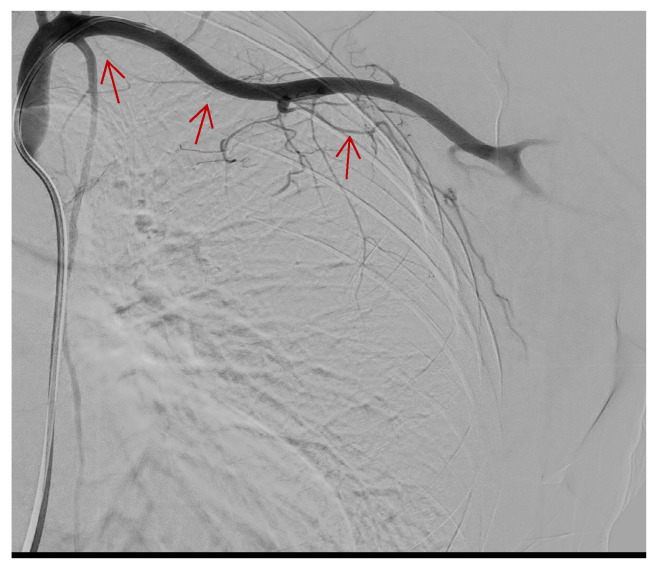
Contrast medium flow in the lateral thoracic artery after selective catheterization of the left subclavian artery. (Red arrows).

**Figure 4:**
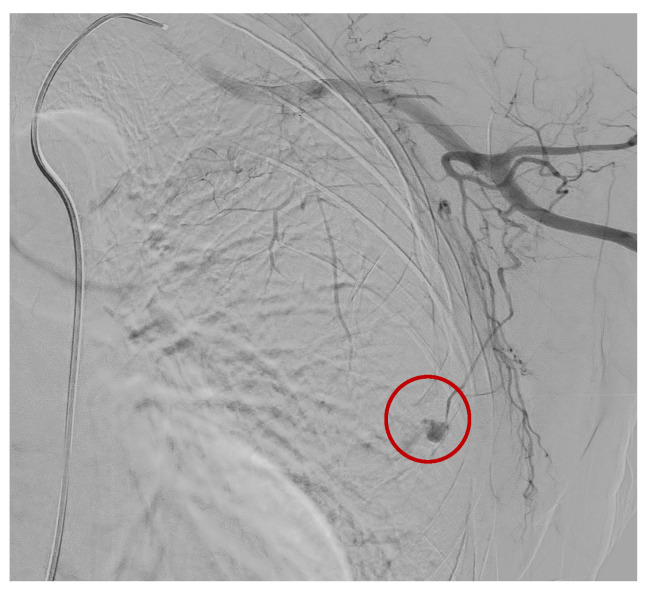
Blood spread by the distal branch of the lateral thoracic artery. (Red circle).

A preliminary angiographic examination documented the spread of a contrast medium by the distal branch of the lateral thoracic artery, which was embolized by releasing two metal coils (2 mm × 2 cm and 2 mm × 4 cm) (Figure 3 and Figure 4).

The final controls confirmed the successful outcome of the procedure with the correct occlusion of the treated vessel without images compatible with active bleeding.

Valid femoral hemostasis was achieved by manual compression. After 3 days of hospitalization, the patient was discharged, and percutaneous drainage of the hematoma was scheduled since she refused open surgery treatment.

Due to the failure of the percutaneous drainage, the patient subsequently accepted the surgical operation, which resulted in an excellent outcome.

The following histopathological analysis was negative: this probably could depend on the few samples collected and the 11 G needle used.

## 3. Discussion

Hematoma is the most frequent post-procedural complication of VABB. Most lesions are centrally distributed where there is an important vascular supply. Kettritz et al. reported that out of 500 patients with microcalcifications, 6 developed a hematoma of at least 4 cm in diameter after this procedure [4,18].

Furthermore, considering the different needle sizes, a greater propensity for bleeding and hematoma onset was described with 8-gauge needles compared to 11-gauge needles 41.9% vs. 8.4%, *p* < 0.001; 35.5% vs. 16.7%, *p* = 0.029) [19].

The majority of the hematomas described the present minimal/moderate volumes, which could result from inadequate compression and/or dressing and spontaneous resolution in a short period of time and without further interventions, which is the most frequent outcome.

Massive hematoma is a rare occurrence, Zheng et al. [20] estimated an incidence of bleeding and hematoma of 11% after VABB, while Simon et al. [21] reported that in 7% of patients, bleeding could not be controlled with standard compression techniques.

A recent study [22] evaluated the efficacy of the thrombin-gelatin matrix as a hemostatic treatment in preventing this frequent procedure-related risk, especially when treating large lesions (>2 cm); the results showed a decreased risk of acute bleeding and a trend of preventing hematoma. An interesting finding is that these complications, as already described by Zheng et al. [20], are independent of the size and number of lesions.

In general, the development of hematoma has been associated with certain factors such as needle size, adjacent vessels, and anticoagulant use [23,24,25,26].

Before proceeding to the description of other such cases, we wanted to emphasize how the literature attested to them as truly rare. A retrospective study in 2019 [27] reported that out of 4776 patients treated, only 0.3% had clinically significant complications, and only 10 cases experienced major hematoma. Moreover, none of this required embolization.

In support of this, there are still a few case reports that highlight this complication, which could present, in addition, different clinical manifestations or causes.

In fact, unlike the case we reported, Nguyen et al. suggested that pseudoaneurysm formation should be suspected when a patient presents with a rapidly growing breast mass [12,28]. On the other hand, clinically, this case was similar; initially, after VABB, a breast ultrasound and standard procedure medication were performed (manual compression for about 10 min and dressing). However, one hour later, the patient felt pain and swelling in the left breast.

Due to the failure of manual compression and injections of tranexamic acid, an ultrasound was repeated, and a pseudoaneurysm was detected. The patient was treated successfully with intravascular embolization [12].

Halicek et al. [23] described a case that initially had no signs referable to active bleeding on evaluation by a Doppler but, after two hours, showed an increase in hematoma volume on ultrasonography and was transferred to the interventional radiology department for further management.

A 3D reconstruction of the CT revealed that the source of the bleeding was due to the perforation of a branch of the left internal thoracic artery. The technically successful coil embolization of the culprit was achieved using 3 mm micro-coils.

Vanni G. et al. [29] reported a patient who was admitted to the Emergency Department for a worsening dry cough, fever (37.5 °C), chest discomfort, feelings of pressure associated with shortness of breath, fatigue, and slight confusion and had undergone a VABB of the left breast four days earlier as a monitoring measure due to a microcalcification cluster localized at the external-superior quadrant.

Despite not experiencing any immediate complication at the time of the procedure, the patient reported progressive swelling since post-procedure day 1.

Open hematoma evacuation and Argon Beam Coagulator hemostasis were performed successfully.

Fishman [30] et al. described two other cases that required embolization to control ongoing bleeding.

Intravascular embolization, using gelfoam, coils, or other devices, is recommended for clinically significant bleeding or hematoma.

Open surgery is to be considered a last resource in case of the failure of the other methods [31].

In our case, a vascular complication occurred by treating an area of microcalcifications with an 11 G needle. Following the failure of the standard compression and dressing, emergency endovascular embolization became necessary. After a few weeks, surgical evacuation was performed on the patient for hematoma resolution and to reduce the infection risk.

## 4. Conclusions

In general, VABB, under ultrasonography or mammography guidance, is evaluated as a minimally invasive, safe, and accurate procedure with low complication risks, although local breast vessel involvement is always a threat.

As we have seen, the cases described presented different clinical pictures and especially different timelines in their development. This makes it difficult to draft an appropriate management protocol, given the heterogeneity of the cases. What we will attempt to do with this study is to add further emphasis to the topic and propose some of the points which are essential for us when performing this type of intervention.

Our patient had no history of coagulopathy, which demonstrates the risk of aggressive vacuum assist, even in patients with normal coagulation and a silent past medical history.

For this reason, it is important to draw up an adequate protocol for the correct management of the patient in the pre- and post-procedural phase; in this case, the protocol applied should follow:

1. The collection of medical history data and informed consent.
2. Check blood chemistry: in particular, hemoglobin, fibrinogen, platelets, prothrombin time, and partial thromboplastin time.
3. Check active pharmacotherapy (antiplatelet, anticoagulant).
4. Check for any allergies.
5. Address peripheral venous catheter positioning.
6. Monitor the patient for at least 60 min after VABB.

The peripheral venous catheter is always positioned by us before this procedure to have quick and ready venous access that can be used in the case of complications, such as anaphylaxis, bleeding, or, more generally, all situations where it might be more complex to find one in time (for example, even low venous compliance related to the patient’s physiology).

In the case of anticoagulant and antiplatelet drugs, we would refer to guidelines and the specialist evaluation of individual cases.

We believe that most acute events occur in this time frame (60 min). Further studies are needed to identify a more appropriate range.

A patient with symptoms illustrated previously and a growing mass at the involved area should always be evaluated for vascular injury or iatrogenic pseudoaneurysm.

In this case report, the protocol was observed, and the patient received a prompt diagnosis and was treated successfully with intravascular embolization.

## Data Availability

This study used open access papers available on PubMed or Google Scholar database. All data are consultable in the References section.
